# A semi–supervised tensor regression model for siRNA efficacy prediction

**DOI:** 10.1186/s12859-015-0495-2

**Published:** 2015-03-13

**Authors:** Bui Ngoc Thang, Tu Bao Ho, Tatsuo Kanda

**Affiliations:** 1 School of Knowledge Science, Japan Advanced Institute of Science and Technology, 1-1 Asahidai, Nomi, Ishikawa Japan; 2 University of Engineering and Technology, Vietnam National University Hanoi, 144 Xuan Thuy, Cau Giay, Hanoi Vietnam; 3 John von Neumann Institute, Vietnam National University Ho at Chi Minh City, Quarter 6, Linh Trung Ward, Thu Duc District, Ho Chi Minh, Vietnam; 4 Graduate School of Medicine, Chiba University, 1-8-1 Inohahan, Chuo-ku, Chiba Japan

**Keywords:** RNAi, siRNA, siRNA design rule, Tensor, Bilinear tensor regression, Semi–supervised learning

## Abstract

**Background:**

Short interfering RNAs (siRNAs) can knockdown target genes and thus have an immense impact on biology and pharmacy research. The key question of which siRNAs have high knockdown ability in siRNA research remains challenging as current known results are still far from expectation.

**Results:**

This work aims to develop a generic framework to enhance siRNA knockdown efficacy prediction. The key idea is first to enrich siRNA sequences by incorporating them with rules found for designing effective siRNAs and representing them as enriched matrices, then to employ the bilinear tensor regression to predict knockdown efficacy of those matrices. Experiments show that the proposed method achieves better results than existing models in most cases.

**Conclusions:**

Our model not only provides a suitable siRNA representation but also can predict siRNA efficacy more accurate and stable than most of state–of–the–art models. Source codes are freely available on the web at: http://www.jaist.ac.jp/\~bao/BiLTR/.

## Background

RNA interference (RNAi) is a cellular process in which RNA molecules inhibit gene expressions, typically by causing the destruction of mRNA molecules. Long double stranded RNA duplex or hairpin precursors are cleaved into short interfering RNAs (siRNAs) by the ribonuclease III enzyme Dicer. The siRNAs are sequences of 19–23 nucleotides (nt) in length with 2 nt overhangs at the 3 ^′^ ends. Guided by RNA induced silencing complex (RISC), siRNAs bind to their complementary target mRNAs and induce their degradation.

In 2006, Fire and Mello received the Nobel Prize for their contributions to research on RNA interference (RNAi). Their work and those of others on discovery of RNAi have had an immense impact on biomedical research and will most likely lead to novel medical applications [1-6]. In RNAi research, highly effective siRNAs can be synthesized to design novel drugs for viral-mediated diseases such as influenza A virus, HIV, hepatitis B virus, RSV viruses, cancer disease and so on. As a result, siRNA silencing is considered one of the most promising techniques in future therapy and predicting their inhibition efficiency is crucial for proper siRNA selection. Therefore finding the most effective siRNAs constitutes a huge challenge facing researchers [7-14]. Numerous algorithms have been developed to design and predict effective siRNAs. These algorithms could be divided into two following generations [15-17].

The first generation consists of siRNA design rule–based tools that were developed through the analysis of small datasets. Various siRNA design rules have been found by empirical processes since 1998. The first rational siRNA design rule was detected by Elbashir *et al.* [18]. They suggested that siRNAs having 19–21 nt in length with 2 nt overhangs at the 3 ^′^ ends can efficiently silence mRNAs. Scherer *et al.* [19] reported that the thermodynamic properties to target specific mRNAs are important characteristics. Soon after these studies, many rational design rules for effective siRNAs have been proposed [20-26]. For example, Reynolds *et al.* [22] analyzed 180 siRNAs systematically, targeting every other position of two 197 −base regions of luciferase and human cyclophilin B mRNA (90 siRNAs per gene), and found the following eight criteria for improving siRNA selection: (i) G/C content 30 −52%, (ii) at least 3 As or Us at positions 15 −19, (iii) absence of internal repeats, (iv) an A at position 19, (v) an A at position 3, (vi) an U at position 10, (vii) a base other than G or C at position 19, (viii) a base other than G at position 13.

However, the performance of tools in the first generation was not high enough to our satisfaction. About 65% of siRNAs produced by the above-mentioned design rules have failed when experimentally tested, says, they were 90% in inhibition and nearly 20% of them were found to be inactive [27]. One reason is that the previous empirical analyses were only based on small datasets and focused on siRNAs for specific genes. Therefore, each of these rules is poor to individually design highly effective siRNAs.

The second generation consists of predictive models by employing machine learning techniques that were learned through larger datasets. Tools based on these models in this generation are more accurate and reliable than tools in the first one [28]. In particular, Huesken and colleagues [29] developed a new algorithm, Biopredsi, by applying artificial neural networks to a dataset consisting of 2431 scored siRNAs (i.e., siRNAs whose knockdown efficacy (score) was experimentally observed). This dataset was widely used to train and test other predictive models such as the ThermoComposition21 [28], DSIR [7], i–Score [15] and Scales models [30]. The five above mentioned models are currently estimated as the best predictors [16,30]. Most notably, Qui *et al.* [31] used multiple support vector regression with RNA string kernel for siRNA efficacy prediction, and Sciabola *et al.* [17] applied three-dimension structural information of siRNA to increase predictability of their regression model. Alternatively, several works [32,33] used classification methods on labeled siRNAs which were experimentally labeled in terms of knockdown efficacy.

It is worth noting that most of those methods suffer from some drawbacks. Their performance is still slow and unstable. It can be caused by the following reasons: (i) siRNAs datasets are heterogeneous provided by different groups under different protocols in different scenarios [33,34]. Thus the performance of these models is considerably decreased and changed when they were tested on independent datasets such as the performance of 18 current models tested on three independent datasets [17]. (ii) The performance of machine learning methods also heavily depends on the choice of data representation (or features) on which they are applied. In the previous models, siRNAs were encoded by binary, spectral, tetrahedron, and sequence representations. However, because of siRNA distribution diversity and unsuitable measures based on these siRNA representations, they can be inappropriate to represent siRNAs in order to build a good model for predicting siRNA efficacy.

Our work aims to develop a higher and more stable model to predict the siRNA knockdown efficacy. To this end, we focus on two main tasks: constructing a appropriate representation of siRNA and building a predictive model. In the first task, in order to enrich the representation of siRNAs, available siRNA design rules in the first generation that are considered as prior background knowledge are alternately incorporated to transformation matrices. In the learning process of these transformation matrices, labeled siRNAs collected from heterogeneous courses are used to capture properties of the proposed representation: the natural clustering property of each class and the distribution diversity of siRNAs. A scored siRNA dataset is also employed to ensure that the representation satisfies the smoothness of our predictive model. In the second task, transformation matrices are weighted and used to transform each siRNA to the enriched matrix representation. A bilinear tensor regression model is developed and learned to predict siRNA knockdown efficacy. To improve the accuracy of the proposed model, the labeled siRNAs are also used in addition to the scored dataset to supervise the learning process of parameters. To obtain more precise data representation, the transformation matrices and parameters are iteratively and simultaneously learned. In the objective function, the Frobenius norm is appropriately replaced by *L*_2_ regularization norm for an effective computation. The contributions of this work are summarized as follows

1. Construct a suitable representation of siRNAs, enriched matrix representation, by incorporating available siRNA design rules and employing both of labeled and scored siRNAs.
2. Develop a higher and stable predictive method to predict the siRNA efficacy by building the bilinear tensor regression model. The learning processes of transformation matrices and parameters of the model are combined together to make more accurate and precise siRNA representation. Labeled siRNAs are used to supervise the learning process of parameters.
3. Quantitatively determine positions on siRNAs where nucleotides can strongly influence inhibition ability of siRNAs.
4. Provide guidelines based on positional features for generating highly effective siRNAs.

We developed a bilinear tensor regression predictor, BiLTR, by using C++ programming language on X–Code environment. BiLTR is experimentally compared with published models on the Huesken dataset and three independent datasets commonly used by the research community. The results show that the performance of the BiLTR predictor is more stable and higher than that of other models.

## Results

This section presents experimental evaluation by comparing the proposed method of bilinear tensor regression model (BiLTR) with the most recent reported methods for siRNA knockdown efficacy prediction on commonly datasets.

The experiments are carried out using four scored datasets

- The Huesken dataset of 2431 siRNA sequences targeting 34 human and rodent mRNAs, commonly divided into the training set HU_train of 2182 siRNAs and the testing set HU_test of 249 siRNAs [29].
- The Reynolds dataset of 240 siRNAs [22].
- The Vicker dataset of 76 siRNA sequences targeting two genes [35].
- The Harborth dataset of 44 siRNA sequences targeting one gene [36].

To construct siRNA representation and learn BiLTR model, we employed labeled and scored siRNA datasets as well as seven siRNA design rules. The seven design rules used to enrich representation of siRNAs are Reynolds rule, Uitei rule, Amarzguioui rule, Jalag rule, Hsieh rule, Takasaki rule and Huesken rule [20-23,29,37,38]. To capture the natural clustering and the diversity properties of siRNAs, and also supervise the parameter learning process, the labeled siRNAs were collected from the siRecords database [27] consisting of siRNAs classified into 4 classes: ‘very high’, ‘high’, ‘medium’, and ‘low’ knockdown efficacy. This database is an extensive one of mammalian RNAi experiments with consistent efficacy ratings. siRecords consists of the records of all kinds of siRNA experiments conducted with various laboratory techniques and experimental settings. In our work, sense siRNAs of 19 nucleotides in length were collected. After removing duplicative siRNAs, ‘very high’ and ‘medium’ and ‘low’ siRNAs were used (to improve the balance between classes while keeping the separation between them, ‘medium’ and ‘low’ siRNAs were merged into one class, denoted by ‘low’). As a result, there are 2470 labeled siRNAs in the ‘very high’ class and 2514 labeled siRNAs in the ‘low’ class. Scored siRNAs in the Huesken dataset were also used to learn BiLTR model.

Transformation matrices *T*_*k*_(*k*=1,…,*K*), coefficient vetors *α* and *β* are learned by employing Algorithm 1. In this algorithm, the convergence criteria were set as follows: the thresholds *ε*, *ε*_1_ and *ε*_2_ were set by small numbers, actually 0.001. The maximum iterative step, *t*_*Max*_, was 2000. Moreover, one crucial issue is to find turning parameters of objective function 10. In our work, the turning parameters of the objective function *λ*_1_, *λ*_2_ and *λ*_3_ were estimated by minimizing a risk function of the proposed model when the model is tested on validation sets. Particularly, besides using the labeled siRNAs and siRNA design rules, we implement 10–fold cross validation on a scored siRNA training set for each turning parameter belonging to the interval [0, log(10)]. The model is trained for each triple of (*λ*_1_, *λ*_2_, *λ*_3_). After that, we compute the following risk function

(1) $$\begin{array}{*{20}l} R(\lambda_{1},\lambda_{2}, \lambda_{3})= \frac{1}{F}\sum_{i=1}^{F}\frac{1}{\parallel {fold}_{i}\parallel}L(T_{1},\ldots,T_{K},\alpha,\beta)  \end{array} $$

where *f* *o* *l* *d*_*i*_ is the validation set, *F* is the number of folds to do cross validation on the training set. *L*(*T*_1_,…,*T*_*K*_,*α*,*β*) is the objective function mentioned in the Methods section. We employ 10-fold cross validation, and thus *F* equals to 10. Concerning the stability of learning turning parameters, 10 times of 10–fold cross validation are implemented. As as result, the fitted turning parameters of each run of 10–fold cross validation are shown in Table 1. Standard deviations of the parameters *λ*_1_, *λ*_2_ and *λ*_3_ are 0.004, 0.00003, and 0.035, respectively so learned turning parameters are more stable. The triple of turning parameters that the value of the risk function is mimimum are employed to learn the final model.

**Table 1:** **The fitted turning parameters of objective function** 10 **in 10 times of 10–fold cross validation**

***λ***_**1**_	***λ***_***2***_	***λ***_***3***_
0.00995033	0.000119984	1.03
0.00995033	0.000119984	1.02
0.00995033	0.000119993	1.03
0.00995033	0.000119993	1.03
0.0198026	0.000119993	1.03
0.0198026	9.9995e-05	1.03
0.00995033	0.00013999	1.03
0.00995033	0.000179984	1.03
0.00995033	0.000179984	1.03
0.00995033	0.000179984	0.92

After finding turning parameters, the final model, BiLTR, is learned by using all of the labeled siRNAs, the siRNA design rules, and the scored siRNA training set.

The BiLTR model is compared to most of state-of-the-art methods for siRNA knockdown efficacy prediction recently reported in the literature. For a fair comparison, we carried out experiments on BiLTR in the same conditions as they did and then compared our obtained results with the ones published in their reports. Concerning training dataset, besides all of models were trained on the same scored siRNA dataset, we also used siRNA design rules and a labeled siRNA dataset to train the BiLTR model. Concretely, the comparative evaluation is as follows

1. Comparison of BiLTR with Multiple Kernel Support Vector Machine proposed by [31]. The authors reported their Pearson correlation coefficient (R) of 0.62 obtained by 10–fold cross validation on the whole Huesken dataset. The Pearson correlation coefficient (R) is carefully evaluated by BiLTR by 10 times of 10-fold cross validation with the average value of 0.64 (Table 2). Concerning the standard deviation (SD) of error rates between predicted and target labels, the SD of our model is 0.23, however Qui and co-workers [31] did not show.

**Table 2:** **The R values and standard deviations of models on the the whole Huesken dataset and HU_test dataset**

**Algorithm**	**Huesken dataset**	**HU_test**
	**(2431 siRNAs)**	**(249 siRNAs)**
Qui’s method	0.62 (–)	–
BIOPREDsi	–	0.66 (0.216)
Thermocomposition21	–	0.66 (0.216)
DSIR	–	0.67 (0.161)
SVM	–	0.80 (–)
**BiLTR**	**0.64 (0.23)**	**0.67 (0.164)**

The Person correlation coefficients R and standard deviations SD are formed by R (SD).
2. Comparison of BiLTR with BIOPREDsi [29], Thermocomposition21 [28], DSIR [7], and SVM [17] when trained on the same scored siRNA dataset, HU_train and tested on the HU_test dataset. The R values of those four models are 0.66, 0.66, 0.67 and 0.80, respectively. The SD values of the first three models are 0.216, 0.216, and 0.161, respectively. However, SD value of the SVM model was not shown. The R value of BiLTR estimated on the HU_test set is 0.67 that is equivalent to the R value of DSIR model, slightly higher than that of the first two models but lower than that of the last model (Table 2). The SD value of the BiLTR model is 0.164 that is similar to the SD value of the DSIR model and higher than that of first two models as well. It can be observed that the performance of SVM is significantly better than that of BiLTR in Table 2.One reason comes from the current limitation of BiLTR as it employs positional features of available design rules but not other characteristics such as GC content, thermodynamic properties, GC stretch, and 3D information while SVM employs positional features and 3D information. This feature captures the flexibility and strain of siRNAs that can be important characteristics for siRNAs of the HU_test set extracted from human NCI–H1299, Hela genes and rodent genes [29]. Therefore, at this moment the performance of the BiLTR model is similar to that of BIOPREDsi, Thermocomposition21, DISR models but cannot achieve higher performance than the SVM model [17] when tested on the HU_test set.
3. Comparison of BiLTR with 18 models including BIOPREDsi, DSIR, SVM when all of models were trained on the HU_train set and tested on three independent datasets of Reynolds, Vicker and Harborth as reported in the recent article [17]. We also computed SD values of error rates between predicted and experimental variables. However, we lack of standard deviations of some models, especially that of the SVM model, because their models’ predicted labels were not shown in their publication. As a result, the BiLTR considerably achieved results higher than all of 18 methods on the all three independent testing datasets as shown in Table 3 (taken from [17] with the last row added for the BiLTR result). The lower performance of SVM than BiLTR in Table 1 can be explained as the added 3D information in SVM does not make it better than BiLTR, especially when testing data are more independent from the Huesken dataset. The lower performance of SVM than BiLTR in Table 3 can be viewed as the added 3D information in SVM does not always make it better than BiLTR, especially when testing data are more independent from the Huesken dataset. Besides that, unlike most of other models, the BiLTR model produces the stable results across each of independent siRNA datasets.

**Table 3:** **The R values and standard deviations of 18 models and BiLTR on three independent datasets**

**Algorithm**	***R*** ^***Reynolds***^	***R*** ^***Vicker***^	***R*** ^***Harborth***^
	**(244si/7 g)**	**(76si/2 g)**	**(44si/1 g)**
GPboot [39]	0.55 (–)	0.35 (–)	0.43 (–)
Uitei [23]	0.47 (–)	0.58 (–)	0.31 (–)
Amarzguioui [20]	0.45 (0.30)	0.47 (0.23)	0.34 (012)
Hsieh [37]	0.03 (0.31)	0.15 (0.23)	0.17 (0.12)
Takasaki [40]	0.03 (0.3)	0.25 (0.23)	0.01 (0.14)
Reynolds 1 [22]	0.35 (0.3)	0.47 (0.224)	0.23 (0.12)
Reynolds 2 [22]	0.37 (0.291)	0.44 (0.232)	0.23 (0.12)
Schawarz [24]	0.29 (–)	0.35 (–)	0.01 (–)
Khvorova [41]	0.15 (–)	0.19 (–)	0.11 (–)
Stockholm 1 [42]	0.05 (–)	0.18 (–)	0.28 (–)
Stockholm 2 [42]	0.00 (–)	0.15 (–)	0.41 (–)
Tree [42]	0.11 (–)	0.43 (–)	0.06 (–)
Luo [43]	0.33 (–)	0.27 (–)	0.40 (–)
i-score[15]	0.54 (0.262)	0.58 (0.19)	0.43 (0.12)
BIOPREDsi [29]	0.53 (0.31)	0.57 (0.23)	0.51 (0.12)
DSIR [7]	0.54 (0.26)	0.49 (0.21)	0.51 (0.11)
Katoh [44]	0.40 (0.34)	0.43 (0.23)	0.44 (0.15)
SVM [17]	0.54 (–)	0.52 (–)	0.54 (–)
**BiLTR**	**0.57 (0.25)**	**0.58 (0.19)**	**0.57 (0.10)**

The Person correlation coefficients R and standard deviations SD are formed by R (SD).

In these comparative studies, it was found that the performance of BiLTR is more stable and higher than that of other models. The first reason is that previous siRNA representations can be unsuitable to represent siRNAs provided different groups under different protocols. In our method, the representation is enriched by incorporating background knowledge of siRNA design rules and learned by employing heterogeneous labeled siRNAs. By combining the representation and parameter learning processes together. Therefore it can capture the distribution diversity of siRNA data. The second reason is that using labeled siRNAs in different distributions to learn our model, BiLTR model can predict more accurate knockdown efficacy of siRNAs.

## Discussion

In this section, we discuss more detail about three main issues: the performance of BiLTR model, the importance of learned transformation matrices and the effect of nucleotide design at particular positions on siRNAs.

Concerning the first issue, as presented in the experimental comparative evaluation, BiLTR achieved better results than most other methods in predicting siRNA knockdown efficacy. There are some reasons for that. First, it is expensive to experimentally analyze the knockdown efficacy of siRNAs, and thus most of available datasets have relatively small size leading to limited results. Second, BiLTR has its advantages by incorporating domain knowledge (siRNA design rules) experimentally found from different datasets. Third, BiLTR is generic and can be easily exploited when new design rules are discovered, or more scored or labeled siRNAs are obtained. As a result, when tested on the three independent datasets generated by different empirical experiments, the performance of BiLTR is better than that of the four above models. Additionally, some models achieve the best results as the BiLTR model when tested on the Vicker dataset (e.g., i-score, Uitei models) but none of them simultaneously reaches the highest result as BiLTR when tested on the three independent datasets (Table 3).

**Figure 1:**
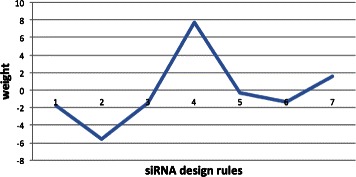
**Contributions of seven siRNA design rule to knockdown ability of siRNAs.**

On the other hand, it is easy to see that the weights *α*_*i*_, *i* = 1, …, *K* show the importance of the siRNA design rules that affect the knockdown efficacy of siRNAs. Figure 1 shows the weights of the seven siRNA design rules. The second and the fourth siRNA ones corresponding to the Uitei and Jalag rules have the smallest and highest weights, respectively. The Uitei rule shows that nucleotides ‘G/C’ at position 1 and ‘A/U’ at position 19 correlate to effective siRNAs and nucleotides ‘A/U’ at position 1 and ‘G/C’ at position 19 correlate to ineffective siRNAs. These characteristics are consistent with most of the other siRNA design rules. However, these characteristics based on positions 1 and 19 are insufficient to generate effective siRNAs. In the fourth rule, except characteristics of the Uitei rule, Jagla and colleagues discovered that effective siRNA have an ‘A/U’ nucleotide at position 10. It also shows the importance of these nucleotides at position 10 when designing effective siRNAs.

**Figure 2:**
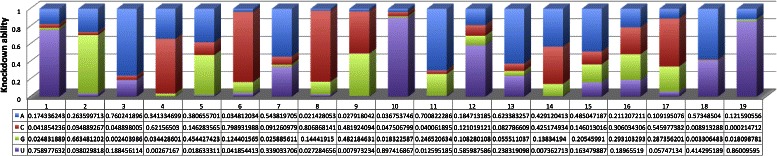
**The learned transformation matrix incorporating positional features of the Reynolds rule.** Histogram shows knockdown efficacy strength of each nucleotide at positions on sense siRNA strand.

Concerning the second issue, the learned transformation matrices not only capture the characteristics of the siRNA design rules but also guide to create new design rules for generating effective siRNA candidates. Table 4 shows the positional features of the Reynolds rule. In this siRNA design rule, effective siRNAs satisfy the following criteria on sense siRNA strands: (i) nucleotide ‘A’ at position 3; (ii) nucleotide ‘U’ at position 10; (iii) nucleotides ‘A/C/U’ at position 13 and (iv) nucleotides ‘A/U’ at position 19. After learning BiLTR, the transformation matrix capturing positional features of the Reynolds rule is determined. Figure 2 shows the learned transformation matrix incorporated with the Reynolds rule. In this figure, each column of the matrix is normalized to easily observe. One of the characteristics is described as “an nucleotide ‘A/U’ at position 19”. This characteristic means that at column 19, the cell (4,19) should contain the maximum value. In the matrix, the value at this cell is 0.86009595 and is the greatest value in this column. We now consider other characteristics of the Reynolds rule. Another characteristic of this rule is that effective siRNAs have at least three nucleotides ‘A/U’ at positions from 15 to 19. In learned transformation matrix, corresponding values of nucleotides ‘A/U’ at positions 15, 18 and 19 are the greatest ones (see Figure 2). Therefore, the transformation matrix can preserve this characteristic of the Reynolds rule. One characteristic of siRNAs such as ‘G/C’ content ranging from 30% to 52% is also preserved in the learned transformation matrix. In addition, positions on siRNAs are not described in characteristics of the design rules, the knockdown efficacy of nucleotides at columns corresponding to these positions are also learned to satisfy the classification assumption and constraints of BiLTR as values at columns 1, 2, 4 and so on. Therefore, after learning the transformation matrices based on the siRNA design rules, these transformation matrices can guide to generate effective siRNAs. For example, Figure 2 shows the Reynolds rule based transformation matrix and its histogram of nucleotides at positions on sense siRNA strand. We can see that effective siRNAs can be designed by using the Reynolds rule and other characteristics such as: ‘U’ at position 12, ‘A’ at position 13, and so on.

**Table 4:** **Characteristics of Reynolds rule**

**Position**	**3**	**10**	**13**	**19**
Effective	A	U	A/C/G	A/U

**Figure 3:**
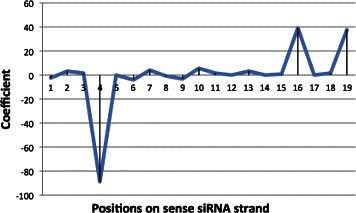
**Coefficients of 19 dimensions corresponding to 19 position on siRNAs.**

Concerning the last issue, we consider the effect of nucleotides at particular positions on siRNAs. In BiLTR model, coefficients *β*_*j*_, *j*=1,…,19, show the strength of the relationship between each variable corresponding to each column of tensors representing siRNAs and the inhibition ability of siRNAs. We know that values of each column show the knockdown efficacy of each nucleotide in a siRNA sequence by incorporating the seven siRNA design rules. Therefore, the coefficients show the influence of nucleotide design at positions on siRNAs to the inhibition ability. In Figure 3, the coefficients at positions 4, 16 and 19 show that the siRNA design at these positions will strongly influence the knockdown efficacy or inhibition of siRNAs. Most of the siRNA design rules also capture the importance of designing nucleotides at positions 16 and 19 but they do not mention the designing of nucleotides at position 4. Therefore, the influence of nucleotides at this position can be considered to design effective siRNAs.

## Conclusion

In this paper, we have proposed a novel method to predict the knockdown efficacy of siRNA sequences by using both labeled and scored datasets as well as available design rules to transform the siRNAs into enriched matrices, then learn a bilinear tensor regression model for the prediction purpose. Besides that, in the model an appropriate siRNA representation is also developed to represent siRNAs belonging to different distributions that are provided by research groups under different protocols.

The experimental comparative evaluation on commonly used datasets with standard evaluation procedure in different contexts shows that the proposed method achieved better results than most existing methods in doing the same task. One significant feature of the proposed method is it can easily be extended when new design rules are discovered as well as more siRNAs are analyzed by empirical processes. By analyzing BiLTR model, we provide guidelines to generate effective siRNAs, and detect positions on siRNAs where nucleotides can strongly effect the inhibition ability.

## Methods

We formulate the problem of siRNA knockdown efficacy prediction as follows

- **Given:** Two sets of labeled and scored siRNAs of length *n*, and a set of *K* siRNA design rules.
- **Find:** A function that predicts the knockdown efficacy of given siRNAs.

Our proposed method consists of three major steps that are described in Table 5.

**Table 5:** **Method for siRNA knockdown efficacy prediction**

1	To encode each siRNA sequence as an encoding matrix *X*representing the nucleotides A, C, G, and U at *n* positions in the sequence. Thus, siRNAs are represented as *n*×4 encoding matrices.
2	To transform encoding matrices by *K* transformation matrices *T*_*k*_ into enriched matrices, *k*=1,…,*K*. Each transformation matrix characterizes the knockdown ability of nucleotides A, C, G, and U at *n* positions in the siRNA sequence regarding the *k*th design rule. Each *T*_*k*_ captures background knowledge of the *k*th design rule. The enriched matrices of size *K*×*n* are considered as second order tensors of the siRNA sequences.
3	To build and learn a bilinear tensor regression model. In this step, *K* transformation matrices as wellas parameters of the model are learned together with the labeled and scored siRNAs and available siRNA design rules. The final model is used to predict the efficacy of new siRNAs.

Step 1 of the method is done where each siRNA sequence with *n* nucleotides in length is encoded as a binary encoding matrix of size *n*×4. In fact, four nucleotides A, C, G, or U are encoded by encoding vectors (1, 0, 0, 0), (0, 1, 0, 0), (0, 0, 1, 0) and (0, 0, 0, 1), respectively. If a nucleotide from A, C, G, and U appears at the *j*th position in a siRNA sequence, *j* = 1,…,*n*, its encoding vector will be used to encode the *j*th row of the encoding matrix.

Step 2 is to transform the encoding matrices by transformation matrices *T*_*k*_ regarding the *k*th design rule, *k*=1,…,*K*. *T*_*k*_ has size of 4×*n* where the rows correspond to nucleotides A, C, G, and U, and the columns correspond to *n* positions on sequences. *T*_*k*_ are learned from the *k*th design rule. Each cell *T*_*k*_[*i*,*j*], *i*=1,…,4, *j* = 1,…,*n*, represents the knockdown ability of nucleotide *i* at position *j* regarding the *k*th design rule. Each transformation matrix has to satisfy types of following constraints. The first type of constraints is basic constraints on elements of *T*_*k*_

(2) $$ T_{k}[i,j]\geq0, \ i=1,\ldots, 4; \ \ j=1,2, \ldots, n   $$

The second type of constraints is generated to incorporate background knowledge of the *k*th siRNA design rule to the transformation matrix *T*_*k*_ (*k*=1,…,*K*). As above mentioned, *T*_*k*_[1,*j*], *T*_*k*_[2,*j*], *T*_*k*_[3,*j*], and *T*_*k*_[4,*j*] show knockdown efficacy of nucleotides A, C, G and U at position *j*th (*j*=1,…,*n*), respectively. Furthermore, the *k*th design rule describes the design of effective siRNAs that consists of the effectiveness or ineffectiveness of nucleotides at some positions of siRNAs. Therefore, trick inequality constraints on the transformation matrix *T*_*k*_ are as follows: in the siRNA design rule *k*th, if some nucleotides at position *j*th are effective, their corresponding values are greater than the other values at column *j*th of *T*_*k*_. In contrast, if some nucleotides are ineffective, their corresponding values are smaller than the other values at column *j*th of *T*_*k*_. For example, the design rule in the right table in Table 6 illustrates that at position 19, nucleotides A/U are effective and nucleotide C is ineffective. It means that the knockdown efficacy of nucleotides A/U are larger than that of nucleotides G/C and knockdown efficacy of nucleotide C is smaller than that of the other nucleotides. Thus, values *T*[1,19],*T*[2,19],*T*[3,19] and *T*[4,19] show the knockdown efficacy of nucleotides A, C, G and U at position 19, respectively. Therefore, five trick inequality constraints at column 19 of *T* are formed. Generally, we denote the set of *M*_*k*_ trick inequality constraints on *T*_*k*_ by siRNA design rule *k*th under consideration by

(3) $$\begin{array}{*{20}l} \{g_{m}(T_{k})<0\}_{m=1}^{M_{k}}  \end{array} $$

**Table 6:** **An example of incorporating the condition of a design rule at position 19 to a transformation matrix** ***T*** **by designing constraints**

**Position**	**Knockdown**	**Nucleotide**	**Mapping**	**Constraints**
	**ability**		**to** ***T***	**on** ***T***
19	Effective	A, U	*T*[1,19],	*T*[3,19]−*T*[1,19]<0
			*T*[4,19]	*T*[3,19]−*T*[4,19]<0
	Ineffective	C	*T*[2,19]	*T*[2,19]−*T*[1,19]<0
				*T*[2,19]−*T*[3,19]<0
				*T*[2,19]−*T*[4,19]<0

where *g*_*m*_(*T*_*k*_)<0 is a trick inequality constraint on transformation matrix *T*_*k*_ that is generated by siRNA design rule *k*th.

Let vector $x_{l}^{(k)}$ of size 1×*n* denote the transformed vector of the *l*th siRNA sequence using the transformation matrix *T*_*k*_. The *j*th element of *x*_*l*_ is the element of *T*_*k*_ at column *j* and the row corresponds to the *j*th nucleotide in the siRNA sequence. To compute $x_{l}^{(k)}$, a new column-wise inner product is defined as follows

(4) $$ \begin{aligned} x_{l}^{(k)}=&T_{k}\circ X_{l}=\left(X_{l}[1,.]T_{k}[.,1], X_{l}[2,.]T_{k}[.,2],\ldots,\right.\\ &\left.X_{l}[n,.]T_{k}[.,n]\right) \end{aligned}  $$

where *X*_*l*_[*j*,.] and *T*[.,*j*] are the *j*th row vector and the *j*th column of the matrix *X*_*l*_ and *T*, respectively, and *xy* is the inner product of vectors *x* and *y*.

Table 7 shows an example of encoding matrix *X*, transformation matrix *T* and transformed vector *x* of the given sequence AUGCU. The rows of *X* represent encoding vectors of nucleotides in the sequence. Given transformation matrix *T* of size 4 × 5. The sequence AUGCU is represented by the vector *x* = (*T*[1,1],*T*[4,1],*T*[3,3],*T*[2,4],*T*[4,5]) = (0.5, 0.1, 0.08, 0.6, 0.1). Therefore, the transformed data can be computed by the column-wise inner product *x*=*T*∘*X*_*l*_.

**Table 7:** **An example of encoding matrix, transformation matrix, and transformed vector (the values 0.5, 0.1 etc. are taken to the vector)**

**Sequence**	**Enconding**	**Transformation**	**Transformed data**
	**matrix** ***X***	**matrix** ***T***	**vector** ***x=T∘X***
AUGCU	1 0 0 0	0.5 0.7 0.32 0.2 0.5	(0.5, 0.1, 0.08, 0.6, 0.1)
	0 0 0 1	0.3 0.1 0.6 *0.6* 0.3	
	0 0 1 0	0.1 0.1 0.08 0.1 0.1	
	0 1 0 0	0.1 *0.1* 0 0.1 *0.1*	
	0 0 0 1		

The third type of constraints relates to preservation of natural clustering properties of each class after being transformed by using transformation matrices *T*_*k*_. It means that siRNAs belonging to the same class should be more similar to each other than siRNAs belonging to the other class. This constraint is formulated as the following minimization problem

(5) $$\begin{array}{*{20}l} &\min \sum_{\substack{p \in N_{1}\\q\in N_{1}}}d^{2}(x_{p}^{(k)},x_{q}^{(k)}) + \sum_{\substack{p \in N_{2}\\q\in N_{2}}} d^{2}(x_{p}^{(k)},x_{q}^{(k)})\\ &\quad-\sum_{\substack{p \in N_{1}\\q \in N_{2}}} d^{2}(x_{p}^{(k)},x_{q}^{(k)})  \end{array} $$

In this objective function, the first two components are the sum of similarities of sequence pairs belonging to the same class and the last one is the sum of similarities of sequence pairs belonging to two different classes; *d*(*x*,*y*) is the similarity measure between *x* and *y* (in this work we use Euclidean distance and *L*_2_ norm); *N*_1_ and *N*_2_ are the two index sets of ‘very high’ and ‘low’ labeled siRNAs, respectively.

In step 3 of the method, each encoding matrix *X*_*l*_ is transformed to *K* representations $(x_{l}^{(1)},x_{l}^{(2)},\ldots,x_{l}^{(K)})$ or (*T*_1_∘*X*_*l*_,*T*_2_∘ *X*_*l*_,…,*T*_*K*_ ∘ *X*_*l*_) by *K* transformation matrices. Denote *R*(*X*_*l*_)=(*T*_1_∘*X*_*l*_,*T*_2_ ∘*X*_*l*_,…,*T*_*K*_ ∘ *X*_*l*_)^*T*^ be the second order tensor of size *K*×*n*. The bilinear tensor regression model can be defined as follows

(6) $$ f(x)= \alpha R(X_{l}) \beta   $$

where *α*=(*α*_1_,*α*_2_,…,*α*_*K*_) is a weight vector of the *K* representations of *X*_*l*_ and *β*=(*β*_1_,*β*_2_,…,*β*_*n*_)^*T*^ is a parameter vector of the model, and *α* *R*(*X*_*l*_) component is the linear combination of representations *T*_1_ ∘*X*_*l*_,*T*_2_∘*X*_*l*_,…,*T*_*K*_∘*X*_*l*_. It also shows the relationship among elements on each column of the second order tensor or each dimension of *T*_*k*_∘*X*_*l*_, *k* = 1,2,…,*K*. Equation (6) can be derived as follows

$$\begin{aligned} f(X_{l})&= \alpha R(X_{l}) \beta = \left(\beta \otimes \alpha^{T}\right)^{T} vec(R(X_{l}))\\ &= \left(\beta^{T}\otimes \alpha \right) vec(R(X_{l})) \end{aligned} $$

where *A*⊗*B* is the Kronecker product of two matrices *A* and *B*, and *v* *e* *c*(*A*) is the vectorization of matrix A.

The fourth type of constraints related to the smoothness and the supervised learning phase of the model by employing labeled siRNAs. An appropriate representation and an accurate model have to satisfy that the knockdown efficacy of each siRNA sequence in the ‘very high’ class has to greater than that of siRNAs in the ‘low’ class. Therefore, let *X*_*p*_ denote the encoding matrix of the *p*th sequence in the ‘very high’ class and *X*_*q*_ denote the encoding matrix of the *q*th sequence in the ‘low’ class. We have the following constraints

(7) $$\begin{array}{*{20}l} \left(f(X_{q})-f(X_{p})\right)&\leq0\Leftrightarrow\alpha \left(R(X_{q})-R(X_{p})\right)\beta\\ &\leq 0\ \ \ p \in N_{1}, q\in N_{2}  \end{array} $$

We see that when labeled siRNAs are collected from heterogeneous courses, these constraints also preserve the stability of model when predicted siRNAs are generated by different protocols.

Therefore, the regularized risk function satisfies the constraints (7) is formulated as follows

(8) $$\begin{array}{*{20}l} L(\alpha,\beta)&=\sum_{l=1}^{N} \left(y_{l}-\alpha R(X_{l})\beta\right)^{2}+\lambda_{1}\parallel \beta^{T}\otimes \alpha\parallel_{Fro}^{2} \\ &\quad+2\lambda_{2}\sum_{\substack{p \in N_{1}\\ q \in N_{2}}}\alpha (R(X_{q})-R(X_{p}))\beta  \end{array} $$

where *λ*_1_, *λ*_2_ are the turning parameters, and ∥*β* ^*T*^⊗*α*∥_*Fro*_ is the Frobenius norm of the first order tensor *β* ^*T*^⊗*α*. *X*_*l*_ and *y*_*l*_ are encoding matrix of the *l*th sequence and its knockdown efficacy in the scored siRNA dataset, and *N* is the size of the scored siRNA sequences. The regularization term in equation (8) is derived as follows

$${\fontsize{8.9}{6}\begin{aligned} \parallel \beta^{T}\otimes \alpha\parallel_{Fro}^{2}&={\sum\nolimits}_{k=1}^{K}{\sum\nolimits}_{j=1}^{n} \left(\alpha_{k}\beta_{j}\right)^{2} = {\sum\nolimits}_{k=1}^{K}{\alpha_{k}^{2}}{\sum\nolimits}_{j=1}^{n} {\beta_{j}^{2}}\\ & = {\sum\nolimits}_{k=1}^{K}{\alpha_{k}^{2}}{\parallel\beta\parallel_{2}^{2}} ={\parallel\alpha\parallel_{2}^{2}}{\parallel\beta\parallel_{2}^{2}} \end{aligned}} $$

Therefore, equation (8) with the Frobenius norm can be replaced by *L*_2_ norm

(9) $$\begin{array}{*{20}l} L(\alpha,\beta)=&\sum_{l=1}^{N} \left(y_{l}-\alpha R(X_{l})\beta\right)^{2}+\lambda_{1}{\parallel\alpha\parallel_{2}^{2}}{\parallel\beta\parallel_{2}^{2}}\\ &+2\lambda_{2}\sum_{\substack{p \in N_{1}\\ q \in N_{2}}}\alpha (R(X_{q})-R(X_{p}))\beta  \end{array} $$

The problem has now become the following multi–objective optimization problem: Finding $\{T_{k}\}_{1}^{K}$, *α* and *β* to minimize objective function (10) under the constraints (2), (3) and minimize objective function (9). The multi–objective optimization problem is equivalent to the following optimization problem.

$$\begin{array}{*{20}l}  &\min\ L(T_{1},\ldots,T_{K},\alpha,\beta)= \sum_{l=1}^{N} \left(y_{l}-\alpha R(X_{l})\beta\right)^{2}\\&\quad+\lambda_{1}{\parallel\alpha\parallel_{2}^{2}}{\parallel\beta\parallel_{2}^{2}}\\ &\quad+\lambda_{2}\sum_{\substack{p \in N_{1}\\ q \in N_{2}}}\alpha (R(X_{q})-R(X_{p}))\beta\\ &\quad+\lambda_{3}\sum_{k=1}^{K}\left(\sum_{p,q \in N_{1}}d^{2}(x_{p}^{(k)},x_{q}^{(k)})+ \sum_{p,q \in N_{2}} d^{2}(x_{p}^{(k)},x_{q}^{(k)})\right.\\ & \quad\left. - \sum_{\substack{p \in N_{1}\\ q \in N_{2}}} d^{2}(x_{p}^{(k)},x_{q}^{(k)})\right) \end{array} $$

Subject to *T*_*k*_[*i*,*j*]≥0, *g*_*m*_(*T*_*k*_) < 0, *i*= 1,…,4;*j*= 1,…,*n*; *k* = 1,..,*K*; *m*= 1,..,*M*_*k*_.

This optimization problem is solved by the following Lagrangian form

(10) $$\begin{array}{*{20}l}  L=&\sum_{l=1}^{N} \left(y_{l}-\alpha R(X_{l})\beta\right)^{2}+\lambda_{1}{\parallel\alpha\parallel_{2}^{2}}{\parallel\beta\parallel_{2}^{2}}\\&+2\lambda_{2}\sum_{\substack{p \in N_{1}\\ q \in N_{2}}}\alpha (R(X_{q})-R(X_{p}))\beta + \sum_{k=1}^{K}\sum_{m=1}^{M_{k}}\mu_{m}^{(k)}g_{m}(T_{k})\\ &+\lambda_{3}\sum_{k=1}^{K}\left(\sum_{p,q \in N_{1}}d^{2}(x_{p}^{(k)},x_{q}^{(k)})+ \sum_{p,q \in N_{2}} d^{2}(x_{p}^{(k)},x_{q}^{(k)}) \right.\\&\left.- \sum_{\substack{p \in N_{1}\\ q \in N_{2}}} d^{2}(x_{p}^{(k)},x_{q}^{(k)})\right)  \end{array} $$

where $\mu _{m}^{(k)},\ m=1,\ldots,M_{k};\ k=1,\ldots,K$ and *λ*_*j*_, *j*=1,…,3 are Lagrangian multipliers. To solve the problem, an iterative method is applied. For each column *j*, *T*_*k*_[.,*j*] is solved while keeping the other columns of *T*_*k*_. *α* and *β* are also solved while keeping the others. The Karush-Kuhn-Tucker conditions are

- Stationarity: $\frac {\partial L}{\partial T_{k}[.,j]}=0,\ \frac {\partial L}{\partial \alpha }=0,\ \frac {\partial L}{\partial \beta }=0,\ i=1,\ldots, 4;\ k=1,\ldots,K; \text {and} j=1, \ldots,n$.
- Primal feasibility: *T*_*k*_[*i*,*j*]≥0, *g*_*r*_(*T*_*k*_)<0, *i*=1,…,4; *j*=1,…,*n*; *r*=1,…,*R*; *k*=1,…,*K*.
- Dual feasibility: $\mu _{m}^{(k)}\geq 0, \lambda _{j}\geq 0,\ m=1, \ldots,M_{k}; \ k=1,\ldots,K; \ j=1,\ldots,3$.
- Complementary slackness: $\mu _{m}^{(k)}g_{m}(T_{k})=0,\ m=1, \ldots,M_{k};\ k=1,\ldots,K$.

From the last three conditions, we have $\mu _{m}^{(k)}=0, \ m=1, \ldots,M_{k};\ k=1,\ldots,K$. Therefore, the stationarity condition can be derived as follows

$${\fontsize{7.5}{6}\begin{aligned} &\frac{\partial L}{\partial T_{k}[.,j]} = \frac{\partial \sum_{l=1}^{N} \left(y_{l}-\alpha R(X_{l})\beta\right)^{2}}{\partial T_{k}[.,j]} +2\lambda_{2}\frac{\partial \sum_{\substack{p \in N_{1}\\ q \in N_{2}}}\alpha (R(X_{q})-R(X_{p}))\beta}{\partial T_{k}[.,j]}\\ &+\lambda_{3}\Bigg(\frac{\partial \sum_{k=1}^{K}(\sum_{p,q \in N_{1}}d^{2}(x_{p}^{(k)},x_{q}^{(k)}) + \sum_{p,q \in N_{2}} d^{2}(x_{p}^{(k)},x_{q}^{(k)})}{\partial T_{k}[.,j]} \\ &- \frac{\partial \sum_{\substack{p \in N_{1}\\ q \in N_{2}}} d^{2}(x_{p}^{(k)},x_{q}^{(k)}))}{\partial T_{k}[.,j]}\Bigg) \\ &=-2\alpha_{k}\beta_{j}\Bigg(\sum_{l=1}^{N} \left(y_{l}-\alpha R(X_{l})\beta\right){X_{l}^{T}}[j,.]+\lambda_{2} \sum_{\substack{p \in N_{1}\\ q \in N_{2}}}(X_{p}[j,.]-X_{q}[j,.])^{T}\Bigg)\\ &+2 \lambda_{3} \sum_{p,q \in N_{1}}(\langle X_{p}[j,.], T_{k}[.,j] \rangle-\langle X_{q}[j,.], T_{k}[.,j] \rangle)(X_{p}[j,.]-X_{q}[j,.])^{T}\\ &+2 \lambda_{3}\sum_{p,q \in N_{2}}(\langle X_{p}[j,.], T_{k}[.,j]\rangle-\langle X_{q}[j,.], T_{k}[.,j] \rangle)(X_{p}[j,.]-X_{q}[j,.])^{T}\\ &-2 \lambda_{3}\sum_{\substack{p\in N_{1}\\q \in N_{2}}}(\langle X_{p}[j,.], T_{k}[.,j]\rangle-\langle X_{q}[j,.], T_{k}[.,j] \rangle)(X_{p}[j,.]-X_{q}[j,.])^{T} = 0 \end{aligned}} $$

Set *Z*_*p*,*q*_=(*X*_*p*_−*X*_*q*_) and set *α*(*R*(*X*_*l*_))_*kj*_ *β*=*α* *R*(*X*_*l*_)*β*−*α*_*k*_ *β*_*j*_ *X*_*l*_[*j*,.]*T*_*k*_[.,*j*]. Therefore, the above formulation is derived as follows

$$\begin{aligned} \frac{\partial L}{\partial T_{k}[.,j]} =& -2\alpha_{k}\beta_{j}\Bigg(\sum_{l=1}^{N} \left(y_{l}-\alpha (R(X_{l}))_{kj}\beta\right){X_{l}^{T}}[j,.] \\ &+\lambda_{2} \sum_{\substack{p \in N_{1}\\ q \in N_{2}}}Z_{p,q}[j,.]^{T}\Bigg)\\ &+2\Bigg(\lambda_{3}\Big(\sum_{p,q \in N_{1}}Z_{p,q}^{T}[j,.]\otimes Z_{p,q}[j,.]\\ &+\sum_{p,q \in N_{2}}Z_{p,q}^{T}[j,.]\otimes Z_{p,q}[j,.]\\ &-\sum_{\substack{p\in N_{1}\\q \in N_{2}}}Z_{p,q}^{T}[j,.]\otimes Z_{p,q}[j,.]\Big)\\ &+{\alpha_{k}^{2}}{\beta_{j}^{2}}\sum_{l=1}^{N}{X_{l}^{T}}[j,.]\otimes {X_{l}^{T}}[j,.]\Bigg)T_{k}[.,j]\\ =&0 \end{aligned} $$

We define the following equations

(11) $$\begin{array}{*{20}l} S(k,j) =&\lambda_{3}\Bigg(\sum_{p,q \in N_{1}}Z_{p,q}^{T}[j,.]\otimes Z_{p,q}[j,.]\\ &+\sum_{p,q \in N_{2}}Z_{p,q}^{T}[j,.]\otimes Z_{p,q}[j,.] \\ &-\sum_{\substack{p\in N_{1}\\q \in N_{2}}}Z_{p,q}^{T}[j,.]\otimes Z_{p,q}[j,.]\Bigg)\\ &+{\alpha_{k}^{2}}{\beta_{j}^{2}}\sum_{l=1}^{N}{X_{l}^{T}}[j,.]\otimes {X_{l}^{T}}[j,.]  \end{array} $$

(12) $$\begin{array}{*{20}l} B(k,j)=& \alpha_{k}\beta_{j}\Bigg(\sum_{l=1}^{N} \left(y_{l}-\alpha (R(X_{l}))_{kj}\beta\right){X_{l}^{T}}[j,.]\\ &+\lambda_{2} \sum_{\substack{p \in N_{1}\\ q \in N_{2}}}Z_{p,q}[j,.]^{T}\Bigg)  \end{array} $$

Substitute equations (11) and (12) to $\frac {\partial L}{\partial T_{k}[.,j]}$, we have

(13) $$ T_{k}[.,j] =S(k,j)^{-1}B(k,j)   $$

$$\begin{array}{*{20}l} \frac{\partial L}{\partial \alpha} =&-2\sum_{l=1}^{N} \left(y_{l}-\alpha R(X_{l})\beta\right)\left(R(X_{l})\beta\right)^{T}\\ &+ 2\lambda_{1} {\parallel\beta\parallel_{2}^{2}}\alpha+2\lambda_{2}\Big(\sum_{\substack{p \in N_{1}\\ q \in N_{2}}} (R(X_{q})-R(X_{p}))\beta\Big)^{T} \\ =&\sum_{l=1}^{N}\alpha\left(R(X_{l})\beta\right)\left(R(X_{l})\beta\right)^{T}-\sum_{l=1}^{N}y_{l}\left(R(X_{l})\beta\right)^{T}\\ &+ \lambda_{1} {\parallel\beta\parallel_{2}^{2}}\alpha  \end{array} $$

(14) $$\begin{array}{*{20}l}  &- \lambda_{2}\beta^{T}\Big(\sum_{\substack{p \in N_{1}\\ q \in N_{2}}} (R(X_{p})-R(X_{q}))\Big)^{T} = 0 \\\ \alpha=&\left(\sum_{l=1}^{N}y_{l}\left(R(X_{l})\beta\right)^{T}+\lambda_{2}\beta^{T}\Big(\sum_{\substack{p \in N_{1}\\ q \in N_{2}}} (R(X_{p})-R(X_{q}))\Big)^{T}\right) \\ &\times \left(\sum_{l=1}^{N}\left(R(X_{l})\beta\right)\left(R(X_{l})\beta\right)^{T}+ \lambda_{1}{\parallel\beta\parallel_{2}^{2}}I\right)^{-1}  \end{array} $$

(15) $$\begin{array}{*{20}l} \frac{\partial L}{\partial \beta} =&-2\sum_{l=1}^{N} \left(y_{l}-\alpha R(X_{l})\beta\right)\left(\alpha R(X_{l})\right)^{T} + 2\lambda_{1} {\parallel\alpha\parallel_{2}^{2}}\beta\\& +2\lambda_{2}\Big(\sum_{\substack{p \in N_{1}\\ q \in N_{2}}} \alpha(R(X_{q})-R(X_{p}))\Big)^{T}  \\ =&\sum_{l=1}^{N}\alpha R(X_{l})\beta \left(\alpha R(X_{l})\right)^{T}-\sum_{l=1}^{N}y_{l}\left(\alpha R(X_{l})\right)^{T} \\&+ \lambda_{1}{\parallel\alpha\parallel_{2}^{2}} \beta -\lambda_{2}\Bigg(\alpha\sum_{\substack{p \in N_{1}\\ q \in N_{2}}} (R(X_{p})-R(X_{q}))\Bigg)^{T}  \\ =&\sum_{l=1}^{N}\left(\left(\alpha R(X_{l})\right)^{T}\otimes\left(\alpha R(X_{l})\right)\right)\beta-\sum_{l=1}^{N}y_{l}\left(\alpha R(X_{l})\right)^{T} \\ &+ \lambda_{1}{\parallel\alpha\parallel_{2}^{2}} \beta \\ &-\lambda_{2}\Bigg(\alpha\sum_{\substack{p \in N_{1}\\ q \in N_{2}}} (R(X_{p})-R(X_{q}))\Bigg)^{T} =0  \\ \beta=&\left(\sum_{l=1}^{N}\left(\left(\alpha R(X_{l})\right)^{T}\otimes\left(\alpha R(X_{l})\right)\right)+ \lambda_{1}{\parallel\alpha\parallel_{2}^{2}}I\right)^{-1}  \\ & \times\!\! \left(\!\sum_{l=1}^{N}y_{l}\left(\alpha R(X_{l})\right)^{T}\,+\,\lambda_{2}\Big(\!\alpha\!\!\sum_{\substack{p \in N_{1}\\ q \in N_{2}}} (R(X_{p})-R(X_{q}))\Big)^{T}\right)  \end{array} $$

The learning phase of the proposed bilinear tensor regression model is summarized in Algorithm 1. In this algorithm, transformation matrices *T*_*k*_,*k*=1,…,*K*, coefficient vectors *α* and *β* are learned together. In particular, siRNA sequences are first represented as encoding matrices. The transformation matrices *T*_*k*_ are initialized following trick inequality constraints generated by siRNA design rule *k*th. Vectors *α* and *β* are also initialized. To learn transformation matrices *T*_*k*_, elements in each column of these matrices are calculated by equation (13). If they satisfy the trick inequality constraints, that column will be updated to the next solution. To learn coefficients of the proposed model, vectors *α* and *β* are updated by equations (14) and (15). The transformation matrices, vectors *α* and *β* are updated until meeting the convergence criteria, where *t*_*Max*_ denotes the maximum iterative step to update *α* and *β*, and *ε*, *ε*_1_ and *ε*_2_ are thresholds for the transformation matrices, vectors *α* and *β*, respectively.


